# The complete genome of *Ziziphus jujuba* cv. dongzao, an economic crop in Yellow River Delta of China

**DOI:** 10.1080/23802359.2017.1383206

**Published:** 2017-09-29

**Authors:** Chun-Ming Gao, Ying-Chun Gao, Xue-Hong Liu

**Affiliations:** a School of Biotechnology, Binzhou University, Binzhou, Shandong, P. R. China;; b Shandong Provincial Key Laboratory of Eco-Environmental Science for Yellow River Delta, Binzhou University, Binzhou, Shandong, P. R. China;; c Shandong Provincial Engineering and Technology Research Center for Wild Plant Resources Development and Application of Yellow River Delt, Binzhou University, Binzhou, Shandong, P. R. China;; d BinZhou Municipal Bureau of Agriculture, Binzhou, Shandong, P. R. China

**Keywords:** Chloroplast genome, *Ziziphus jujuba* cv. dongzao, Yellow River Delta

## Abstract

The complete chloroplast genome of *Ziziphus jujuba* cv. dongzao, known as an important economic cultivar in Yellow River Delta of China was reported. It exhibits a quadripartite structure with 161,493 bp including a large single copy region (89,178 bp), a small single copy region (19,357 bp) and a pair of inverted repeats regions (26,479 bp). It has 36.79% GC content and 114 unique genes. Phylogenetic analysis showed that it was a member of *Ziziphus* and more closely related to *Berchemiella wilsonii.* Border analysis revealed that there were some differences in the borders of the four related cultivars.

The Yellow River Delta, covering about 6010 km^2^ in southwestern coast of Bohai Sea in China, has become the youngest coastal wetland ecosystem (Xu et al. 2004; Li et al. 2016). But, soil salinization has always existed in this area (Wang et al. 2017). *Ziziphus jujuba* Mill. cv.dongzao, as an important saline-alkali tolerant plant has become the most popular and highly valued fruit tree in Yellow River Delta (Yan and Gao 2002; Zhang et al. 2016). In this study, the complete chloroplast genome of *Z. jujuba* cv.dongzao was sequenced and analyzed, aiming for further genetic study.

The sample of *Z. jujuba* cv.dongzao was collected in Binzhou, Shandong province of China, with the voucher (BZG2016009) deposited in Binzhou University. Total genomic DNA was extracted and sequenced following the method of Yang et al. (2014) at Kunming Institute of Botany, Chinese Academy of Sciences. The complete cp genome was acquired in Geneious v8.1 (Kearse et al. 2012). The annotation was conducted using DOGMA (Wyman et al. 2004). The genome map was illustrated with the help of CPGAVAS (Liu et al. 2012) and the annotated sequence was submitted to NCBI (MF781071).

The complete chloroplast genome of *Z. jujuba* cv.dongzao is 161,493 bp in length. It maps as a typical quadripartite circular structure including a large single copy (LSC) region with 89,178 bp, a small single copy (SSC) region with 19,357 bp and a pair of inverted repeats (IRs) regions with 26,479 bp. It has 36.79% GC content and 114 unique genes consisting of 80 protein-coding genes, 30 tRNA genes and 4 rRNA genes. Six protein-coding genes, 7 tRNA genes and 4 rRNA genes were duplicated in IR regions. In particular, the rps12 was recognized as the trans-spliced gene. Notably, the genes infA, ycf1 were interpreted as pseudogene due to lack of open reading frame (ORF).

Phylogenetic analysis was performed using whole cp genomes of eight species in Rosales by maximum parsimony method in MEGA 6.0 (Tamura et al. 2013). Result showed that *Z. jujuba* cv.dongzao as a member of *Ziziphus* was closely related to *Berchemiella wilsonii* Nakai (BP = 100) (Figure 1(A)). Meanwhile, we compared the borders of LSC, SSC and IR regions in genomes of *Z. jujuba* cv.dongzao (Ma et al. 2017), *Z. jujuba* cv.jinsixiaozao, *Z. jujuba* cv.junzao and *Z. jujuba* var.spinosa (Figure 1(B)). Firstly, the IRa extended into the rps19 gene with a short 107 bp in the four genomes. Then, ycf1 was located entirely in the IRa regions in the genomes of *Z. jujuba* cv.dongzao and *Z. jujuba* cv.jinsixiaozao, which resulted in a pseudogene. But, the SSC regions expanded 8 bp to ycf1 in the others. In addition, the border between IRb and SSC regions was located in the gene ycf1 with the SSC region expanded 11 bp, 20 bp, 4591 bp and 4591 bp to ycf1, respectively, while ycf1 was detected as a pseudogene just in *Z. jujuba* cv.jinsixiaozao. Also, a short pseudogene rps19 was just detected at IRb/LSC border in *Z. jujuba* cv.jinsixiaozao.

**Figure 1:**
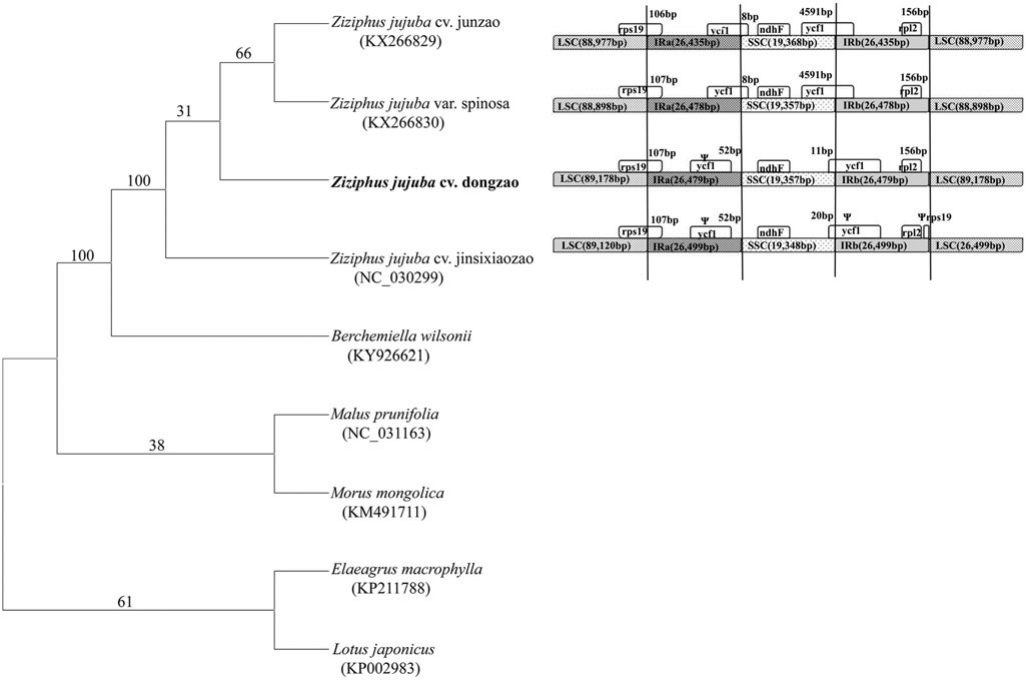
(A) Molecular phylogeny of *Ziziphus jujuba* cv. dongzao and other species in Rosales based on whole cp genome using MEGA 6. (B) Comparison of the borders of LSC, SSC and IR regions among four cultivars in *Ziziphus*.
